# Ultrastructural characterisation of *Bacillus subtilis* TatA complexes suggests they are too small to form homooligomeric translocation pores

**DOI:** 10.1016/j.bbamcr.2013.03.028

**Published:** 2013-08

**Authors:** Daniel Beck, Nishi Vasisht, Jacopo Baglieri, Carmine G. Monteferrante, Jan Maarten van Dijl, Colin Robinson, Corinne J. Smith

**Affiliations:** a School of Life Sciences, University of Warwick, Coventry CV4 7AL, UK; b Department of Medical Microbiology, University of Groningen, University Medical Center Groningen, Groningen, The Netherlands

**Keywords:** Tat translocation, Protein transport, Gram positive bacteria, Electron microscopy, Single particle image processing

## Abstract

Tat-dependent protein transport permits the traffic of fully folded proteins across membranes in bacteria and chloroplasts. The mechanism by which this occurs is not understood. Current theories propose that a key step requires the coalescence of a substrate-binding TatC-containing complex with a TatA complex, which forms pores of varying sizes that could accommodate different substrates. We have studied the structure of the TatAd complex from *Bacillus subtilis* using electron microscopy to generate the first 3D model of a TatA complex from a Gram-positive bacterium. We observe that TatAd does not exhibit the remarkable heterogeneity of *Escherichia coli* TatA complexes but instead forms ring-shaped complexes of 7.5–9 nm diameter with potential pores of 2.5–3 nm diameter that are occluded at one end. Such structures are consistent with those seen for *E. coli* TatE complexes. Furthermore, the small diameter of the TatAd pore, and the homogeneous nature of the complexes, suggest that TatAd cannot form the translocation channel by itself. Biochemical data indicate that another *B*. *subtilis* TatA complex, TatAc, has similar properties, suggesting a common theme for TatA-type complexes from *Bacillus*.

## Highlights

• First 3D density maps of TatA-type complexes from Gram-positive organism
• TatAd forms ring-shaped complexes with potential pores of 2.5–3 nm diameters.
• The small diameter of TatAd channels apparently precludes passage of folded proteins.
• TatAc forms homogeneous complexes of < 100 kDa.
• Similarities between *B. subtilis* TatAd and TatAc suggest a common mechanism.

## 1 Introduction

The twin-arginine translocation (Tat) pathway is an unusual system that acts to translocate fully folded proteins across the bacterial plasma membrane, and the chloroplast thylakoid membrane [1–3]. The Tat system functions in parallel with the well characterised Sec pathway and is dependent on the presence of a proton-motive force across the membrane [4–6]. It derives its name from the highly conserved twin-arginine motif present in the N-terminal signal peptide of Tat substrates [7,8]. In Gram-negative bacteria the integral membrane proteins TatA (10 kDa), TatB (18 kDa) and TatC (30 kDa) comprise the minimal components required for translocation of Tat substrates [9–11], and these proteins are all expressed from a single operon. An additional *tat* gene, *tatE*, is expressed elsewhere in the genome of *Escherichia coli*. The *tatE* gene is thought to be a cryptic duplication of *tatA* due to its high degree of sequence similarity [9] and ability to functionally complement a *ΔtatA* mutant [12].

Under steady state conditions the TatABC core components are observed to form two primary types of integral membrane complexes: a TatABC substrate-binding complex of ~ 370 kDa and TatA complexes ranging in size from 50 kDa to over 500 kDa [13–15]. The TatABC complex and TatA complex or complexes are thought to transiently coalesce to form the active translocon, and TatA has been suggested to form pores of varying sizes that could accommodate different substrates. Recent studies on the function of TatC have refined this model, suggesting a role for TatC in inserting the twin-arginine signal sequence into the membrane and a combined role for TatB and TatC in substrate recognition [16,17]. In addition, information from a recent crystal structure of TatC suggests that TatA could bind within a large, concave face of Tat C [16,17]. Low resolution structures of purified TatA suggest that this protein assembles into ring-shaped particles of 9–13 nm in diameter, a small subset of which have an internal cavity that could be large enough to accommodate the larger Tat substrates [18]. On the other hand, recent investigations have shown that TatE, which can apparently fully substitute for TatA, forms complexes that are much smaller and more homogeneous. These complexes appear as rings of 6–8 nm [12] which are too small to accommodate large Tat substrates in a folded state. The precise nature and function of TatA-type complexes of *E*. *coli* is thus currently unclear.

In contrast to Gram-negative bacteria, almost all Gram-positive bacteria possess a ‘minimalist’ Tat system which lacks a TatB component. Interestingly some Gram-positive organisms contain multiple Tat systems which possess differing substrate specificities [19]. *Bacillus subtilis* is one such bacterium, containing two minimal TatAC-type complexes, termed TatAdCd and TatAyCy, along with a third TatA component, TatAc [20]. The *tatAd* and *tatCd* genes are expressed under phosphate-limiting conditions and are located together in an operon downstream of the *phoD* gene. The PhoD protein has both phosphodiesterase and alkaline phosphatase activity, and is the only known substrate of the TatAdCd translocase [21–23]. The absence of a TatB component is compensated for by the bifunctional role of the TatAd protein, which has been shown to complement both *E*. *coli tatA*/*E* and *tatB* null mutants [24]. As with the *E*. *coli* system, the *B*. *subtilis* Tat components have been shown to form two types of complexes; a TatC-containing complex that we assume to be functionally analogous to the TatABC complex, and a separate TatA complex. However, biochemical assays have suggested that the *B*. *subtilis* complexes are significantly smaller and more homogeneous than the *E*. *coli* versions. The TatAdCd complex runs at ~ 230 kDa during blue-native PAGE and the TatAd complex has been estimated to be ~ 160 kDa by gel filtration [24]. Similarly, both TatAyCy and TatAy have been reported to form ~ 200 kDa complexes as judged by gel filtration [25,26]. The earlier studies also showed that the TatAdCd system is able to export the large, cofactor-containing trimethylamine N-oxide (TMAO) reductase (TorA) substrate (~ 90 kDa) when expressed in an *E*. *coli tat* null mutant. These data therefore suggest that multiple-sized TatA complexes are not essential for the effective transport of Tat substrates. Consistent with these findings, an alternative model for Tat translocation suggests that a localised concentration of TatA components acts to destabilise the lipid bilayer to facilitate transport [27].

Despite the clear functional overlap between the Tat systems of *E*. *coli* and *B*. *subtilis*, the available evidence suggests major differences in complex organisation. In this study we report the first structural investigation of membrane-localised *B*. *subtilis* TatA complexes and present 3D density maps of TatAd complexes obtained through 3D reconstruction of random conical tilt pairs of electron microscopy (EM) images. These data show TatAd complexes to be smaller and more homogeneous than *E*. *coli* TatA complexes, with no indication of a pore large enough to translocate the larger Tat substrates in a folded state. We additionally characterise a small and homogeneous *B*. *subtilis* TatAc complex that also supports efficient translocation of Tat substrates. The clear biochemical and structural differences that we observe challenge current thinking and suggest an alternative mechanism for Tat translocation in *B*. *subtilis*, and possibly *E*. *coli*.

## 2 Experimental procedures

### 2.1 Plasmid construction

Procedures for DNA purification, restriction, ligation, agarose gel electrophoresis, and transformation of competent *E*. *coli* cells were carried out as described by Sambrook et al. [28]. Plasmid DNA from *E*. *coli* was isolated using the alkaline lysis method, or the innuPREP Plasmid small kit from Analytik Jena. To construct the plasmid pBAD-Ac-Strep, a copy of the *tatAc* gene with 3′ sequences encoding a Strep-II tag was PCR-amplified from the genome of *B*. *subtilis* 168. The 5′ primer 5'-GGGCCATGGAATTAAGCTTCAC AAAAATACTCG-3′ contained an *Nco*I restriction site whereas the 3′ primer 5′-GGGTCTAGAC TATTTTTCAAACTGTGGGTGCGACCAATTCGACATTTGTTTGTCTTCTTTGTTTTCTG-3′ contained an *Xba*I restriction site and sequences encoding the Strep-II tag. The resulting PCR-product was cleaved with *Nco*I-*Xba*I and ligated into the *Nco*I-*Xba*I-cleaved pBAD24 plasmid.

### 2.2 Expression and purification of the TatAd complex

*E*. *coli* was grown aerobically in Lysogeny broth (LB). *E*. *coli ΔtatABCDE* cells containing plasmid pBAd-His were grown aerobically to mid-exponential phase (OD_600_ 0.64) before induction of *tatAd* expression using 0.5 mM arabinose. Membranes were isolated as described previously and solubilised in 1% n-dodecyl β-d-maltoside (DDM). Solubilised membranes were added to a 10 ml Talon slurry equilibrated with one column volume of buffer 1 (20 mM Tris–HCl pH 8.0, 400 mM NaCl, 5 mM imidazole and 0.02% DDM) and left rotating overnight at 4 °C. The protein and slurry mix was then poured into a column and allowed to settle for approximately 30 min before elution of the flow through. The Talon column was subsequently washed with 6 column volumes of buffer 1. Bound protein was eluted from the column using a 150 mM imidazole solution of buffer 1 and twenty 1.4 ml fractions were collected. All TatAd-containing fractions, as identified by Western blot, were pooled (~ 20 ml volume) and concentrated using 10 kDa MWCO Vivaspin concentrators to ~ 2 ml final volume.

### 2.3 SDS-Page and Western blotting

Proteins were separated using SDS-polyacrylamide gel electrophoresis and immunoblotted using anti-His IgG, with detection via a secondary anti-mouse IgG horseradish peroxidise conjugate. The EZ-ECL detection kit was used for visualisation.

### 2.4 TMAO reductase activity assay

The TMAO reductase activity assay was performed as described previously [13,29]. *E*. *coli* cells were grown anaerobically until mid-exponential growth phase prior fractionation into periplasmic, cytoplasmic, and membrane fractions. The cell fractions were loaded and separated on a 10% native polyacrylamide gel that was subsequently assayed for TMAO reductase activity as described previously.

### 2.5 Blue native polyacrylamide gel electrophoresis

Blue native polyacrylamide gel electrophoresis was performed as described previously [15]. The membranes were prepared as described above and solubilised in 50 mM Bis–Tris, pH 7.0, 750 mm 6-aminocaproic acid, and 2% (w/v) digitonin. Solubilised membranes were loaded and separated on a polyacrylamide gradient gel (5–13.5%). The proteins were detected by immunoblotting as described above.

### 2.6 Gel-filtration chromatography

A 240 μl sample of the TatAd-containing concentrate was loaded onto a Superdex™ 200 HR 10/30 gel filtration column (GE Healthcare Life Sciences), equilibrated and run with GF buffer 1 (20 mM Trizma, 150 mM NaCl, 0.02% DDM) at a 0.5 ml/min flow rate.

### 2.7 Grid optimisation

All grids were produced using gel filtration fraction 21 ([protein] ~ 24 μg/ml). Dilutions of this fraction were prepared in GF buffer 1 with DDM. All grids were processed using the following protocol: 4 μl of sample was applied to freshly glow-discharged (negatively) carbon-coated copper grids (300 mesh, Agar Scientific) for 1 min. The grids were then washed twice with GF buffer 1 minus DDM for ~ 10 s using the touching drop method [30]. This was followed by staining twice with 2% uranyl acetate for 20 s and then air drying for ~ 10 min before imaging.

### 2.8 Electron microscopy

The samples were imaged using a 200 kV JEOL 2011 field emission gun (FEG) TEM operating a 4 k Gatan Ultrascan CCD camera with a pixel size of 14 μm. Images were obtained at a magnification of ~× 57,000 under low-dose conditions giving 2.62 Å/p. For the tilt pairs, images were collected first at 50°, then 0° at a range of defoci giving 119 initial images. 22 tilt pairs of images were selected for particle picking, with an average defocus of 1.8 μm for the untilted micrographs.

### 2.9 Image processing

The majority of the image processing was performed using Spider/Web software [31] managed through the SPIRE GUI interface [32]. Micrograph quality and defocus of the untilted images were estimated using both CTFFIND3 and the TF ED Spider operation. 2540 particles were picked interactively from 22 tilt pairs using the JWEB pair-wise picking option and a box size of 128 × 128 pixels. CTFTILT3 [33] was used to verify the tilt angle of the tilted images and to calculate the defocus across these images. Particles were normalised and then CTF-corrected by phase-flipping. The untilted particles were iteratively centred using a reference-free method. A cross-correlation check to the rotationally averaged sum image was used to remove badly matching particles. All the remaining particles were assessed for any variation in size following the published method [34]. The dataset was separated into four potential size classes showing an overall variation of ~ 1 nm in diameter across the class averages. To validate the size classification, the centred class averages were used as cross-correlation references for the entire particle set, allowing inter-class movement of particles to a more appropriate fit. This was repeated until inter-class particle movement reached a stable minimum, producing classes containing 386–598 particles. Each class was then aligned rotationally and translationally using a reference-free method. Corresponding tilted images were centred only using an iterative method of custom masking. After defining the Euler angles Φ (in plane rotation, from the untilted particle alignment) and θ (known tilt angle), 3D reconstructions were calculated by back projection using the SIRT algorithm conjugate gradient method [35].

## 3 Results

### 3.1 Overexpression and purification of TatAd

**Fig. 1:**
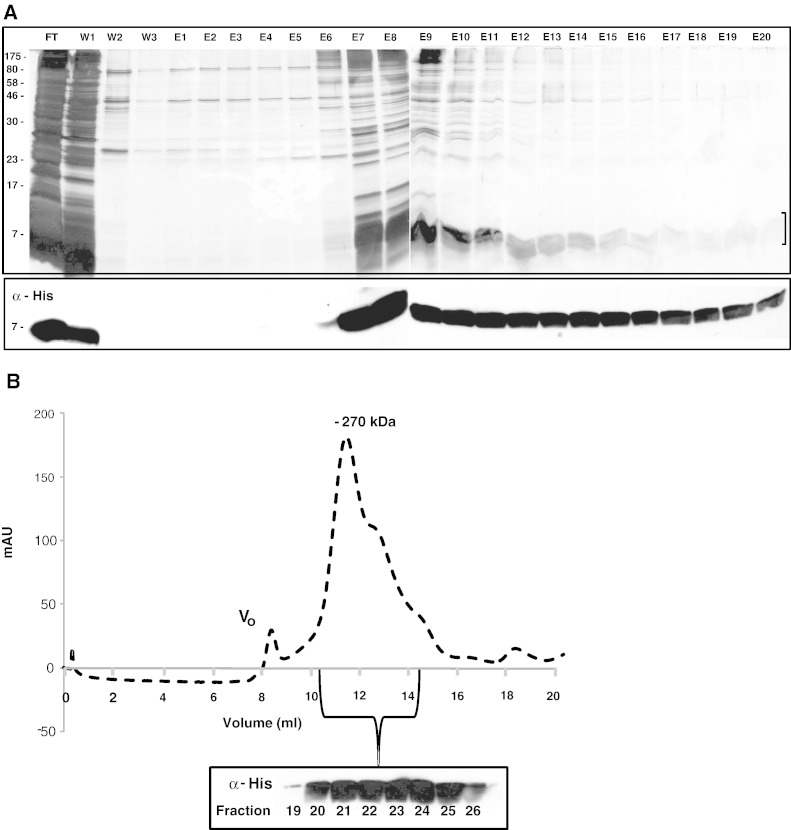

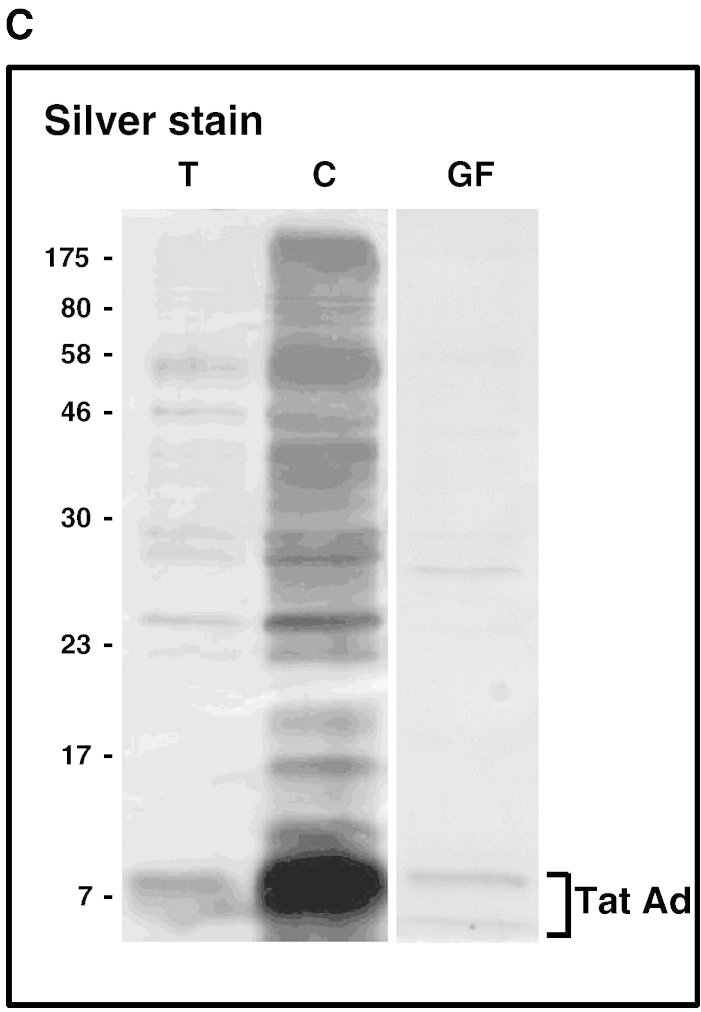
Purification of *B*. *subtilis* TatAd. (A) Membranes were isolated from *E*. *coli Δtat* cells expressing *B*. *subtilis* TatAd with a C-terminal His-tag, solubilised in DDM and applied to a Talon affinity column. The proteins in all elution fractions were separated by SDS-PAGE and the gels were analysed using silver staining or immunoblotting with antibodies against the His-tag. Talon column fractions: FT = flow-through, W1-3 = wash fractions, E1-E20 = elution fractions. The bracket indicates the position of TatAd which runs as a diffuse band. (B). A sample of the TatAd concentrate was applied to a Superdex 200 GL 30/100 gel filtration column. The run (240 μl sample, 0.5 ml/min flow, 0.02% DDM in buffer) shows a major peak at ~ 270 kDa with a shoulder towards lower molecular weights. The corresponding Western blot shows that TatAd elutes across fractions 19–26. V^o^ = void volume. (C) Silver-stained gel of elution fractions which contain TatAd for different stages in column purification. T = pooled Talon column elution fractions, C = pooled Talon fractions after concentration, GF = gel filtration elution fraction selected for electron microscopy studies. Positions of TatAd are indicated on the right of the figure and molecular weight markers are shown on the left.

TatAd was expressed in *E*. *coli ΔtatABCDE* cells from the pBAd-His plasmid. The cells were fractionated and the membranes isolated before solubilisation in 1% DDM and subsequent purification on a Talon-affinity column, as described in the Experimental procedures section. All column fractions were analysed by SDS-PAGE, and visualised by silver stain as well as immunoblotting using antibodies to the C-terminal His-tag on TatAd. As shown in Fig. 1A, a proportion of TatAd was detectable in the initial wash fraction, but the majority of the protein bound well to the column, eluting over fractions E7-E20. TatAd-containing elution fractions were then pooled and concentrated (with no significant loss of Tat protein). A sample of the concentrate was loaded onto a calibrated Superdex^TM^ 200 HR 10/30 gel filtration column (Fig. 1B). TatAd complexes were found to elute with an estimated mass of ~ 270 kDa, as observed previously [24], with a shoulder in the elution profile corresponding to a mass of ~ 160 kDa. These size estimates are greatly influenced by the detergent micelle and, as in previous Tat studies, the true sizes of the complexes are likely to be smaller. All elution fractions were immunoblotted using antibodies to the C-terminal hexahistidine tag on TatAd. The immunoblot shows TatAd to elute across fractions 19–26 which correspond to the major gel filtration peak and its shoulder in the elution profile (Fig. 1B and C). It should be noted that TatAd runs as a diffuse band near the dye front and stains poorly with silver as previously noted [24]. Hence although some contaminating bands are evident, these represent minor contaminants in comparison to TatAd. Ni-NTA-Nanogold® labelling of TatAd via its histidine tag showed association with complexes in the 7–9 nm size range (data not shown).

### 3.2 Single particle analysis, size classification and 3D reconstruction of TatAd complexes

**Fig. 2:**
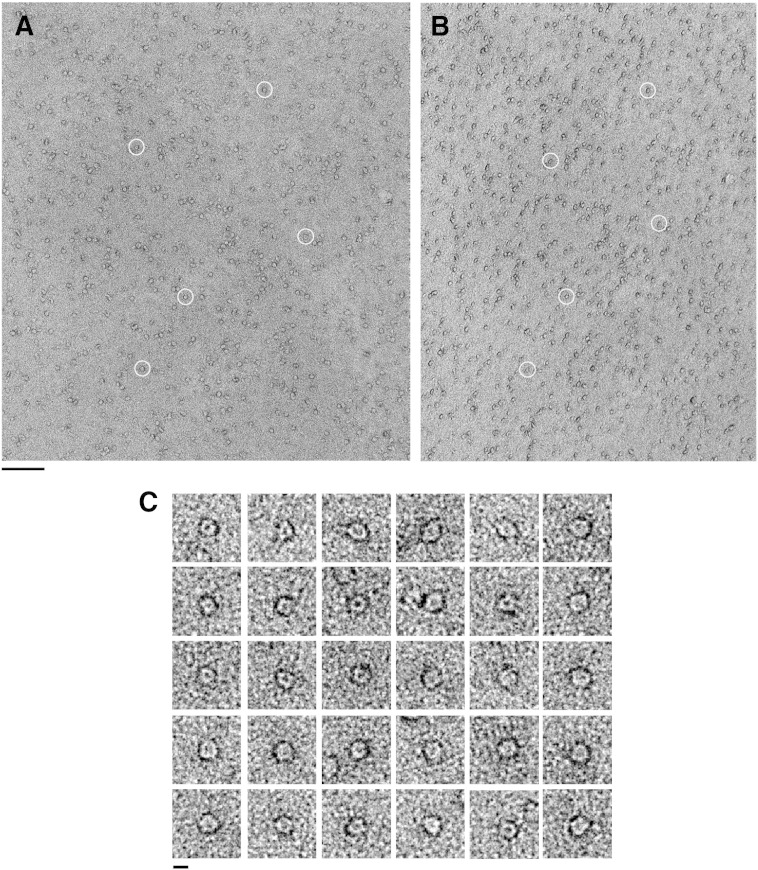
Micrographs of TatAd tilt pair and example particles. The area of micrographs is shown untilted (A) and tilted to 50° (B). Corresponding particle pairs in both images are circled. The scale bar measures 100 nm. Images were acquired at ~ 57,000 × magnification under low-dose conditions. (C) Particles picked from the untilted images are small and roughly circular measuring ~ 7.5 nm in diameter. Each contains a clear central pool of stain indicating a cavity or channel. The scale bar is set to 7.5 nm to facilitate size comparison with the actual particles.

Samples of TatAd complexes were taken from under the major gel filtration peak (fraction 21 in Fig. 1B) and analysed by single particle EM in negative stain. After screening for optimal imaging conditions, it was found that a 1:8 dilution with GF buffer (which contained 0.02% DDM) resulted in a relatively well-dispersed, homogeneous population which, whilst a few particles were clustered in pairs or small groups, enabled more than 2000 single particles of TatAd to be selected for analysis. (Fig. 2A). This dilution was used for the subsequent imaging and single particle analysis. The TatAd particles adopted a preferred orientation on the grid. Therefore, a random conical tilt reconstruction strategy was used for single particle analysis [36], which provides the range of alternative views required to compute a 3D model. Accordingly, micrographs were taken in pairs, both untilted (Fig. 2A) and tilted to 50° (Fig. 2B). TatAd complexes appear as small, discrete and well-defined ring-shaped particles with a central pool of stain (Fig. 2C) similar to those observed previously for TatA and TatE of *E*. *coli*.

**Fig. 3:**
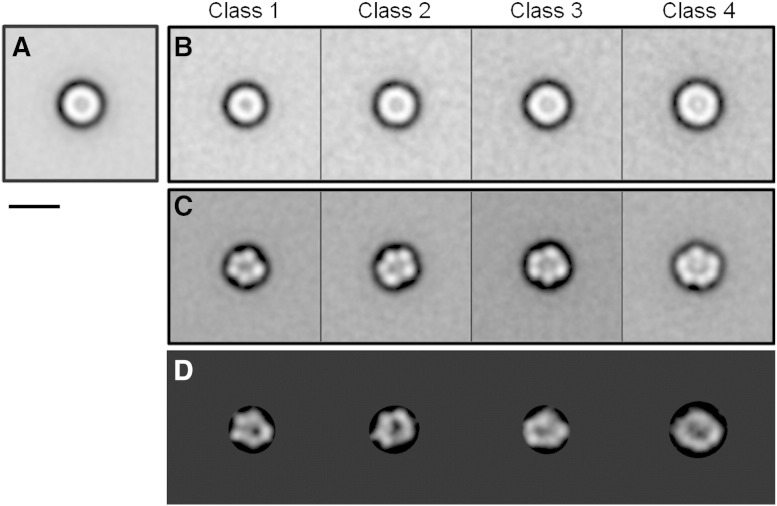
Assessment of size variations in TatAd complexes. (A) Initial centred sum image of 1990 particles. (B) Size class averages of TatAd reveal a slight size variation that correlates with internal density (see largest class, top right). The numbers of particles per class are as follows: Class 1, 547; Class 2, 477; Class 3, 440; Class 4, 352. (C) Size classes after reference-free rotational and translational alignment of the untilted particles. (D) Reprojections of TatAd 3D density maps for comparison to the class averages. Scale bar = 10 nm.

**Fig. 4:**
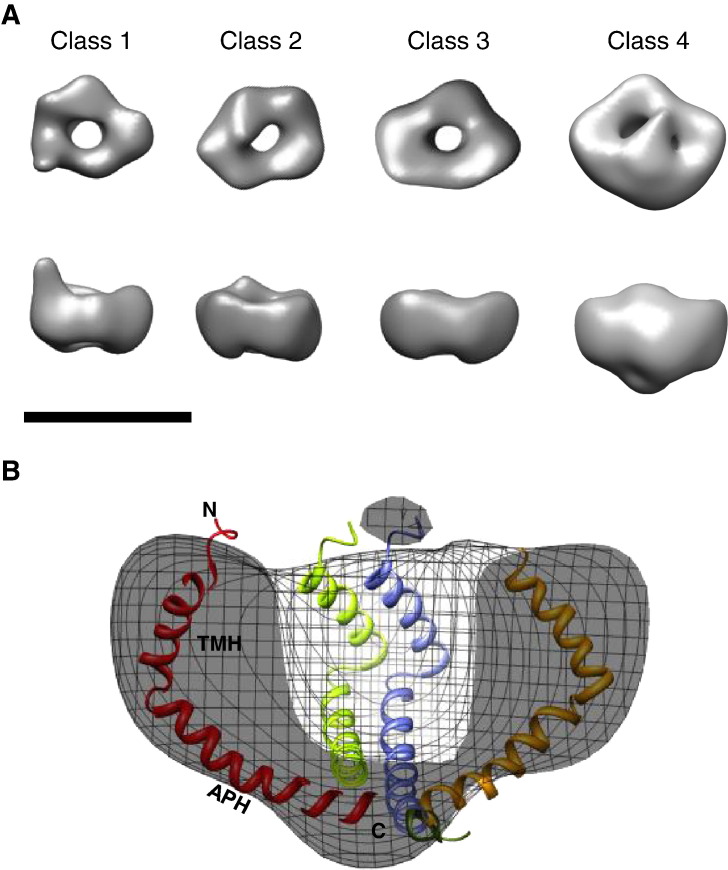
3D density maps of TatAd complexes. (A) Shown from left to right are Classes 1–4. The maps are filtered to 30 Å and contoured at ~ 4σ (standard deviations above the mean density). Scale bar = 10 nm. (B) Cross-section of a Class 4 complex to show the internal cavity. In order to illustrate the compatible size and shape of TatAd monomers with this map, the solution state NMR structures of TatAd have been placed manually into the 3D density. Separate monomers are coloured in red, yellow, green and blue. Side views in (A) and (B) are shown with the potential cytoplasmic side facing down as suggested by the fitted TatAd NMR structure. TMH, transmembrane helix; APH, amphipathic helix.

A total of 2540 initial particles were picked interactively from across 22 tilt pairs of micrographs. All particles were normalised and corrected for the effect of the contrast transfer function (CTF) by phase flipping. After discarding particles which were clipped or broken, 1990 untilted particles were iteratively centred using a reference-free method. This gave an initial rotationally averaged sum image of ~ 7.5 nm diameter (Fig. 3A). The particles were then assessed for size variation following the published method [34]. Using multivariate-statistical analysis (MSA), the dataset was separated into four potential size classes showing an overall variation of only ~ 1.5 nm in diameter (Fig. 3B). This initial size classification was validated using a multi-reference alignment and re-classification method (see Experimental procedures section). The smallest class measures only 7.5 nm in diameter (Class 1) and is the most abundant (547 images) and well defined. The largest class is 9 nm in diameter (Class 4) and is the least populated (352 images). Attempts were made to identify larger rings from within the discarded particles, but no significant population could be found. A variation in the intensity of central staining that correlates with the diameter of the particle was also observed. Clear central density is present in the Class 4 average whilst this is absent in Class 1. After reference-free alignment of the untilted particles (Fig. 3C) and centring of the corresponding tilted particles, initial 3D models were generated. These density maps were then iteratively refined by matching the original tilted particles to projections of the initial models to improve their cross-correlation. In Fig. 3D the class averages are compared to reprojections of their corresponding final density map. In Classes 1–3, the 3D density map appears as a small asymmetrical ring made up of 5 globular densities measuring ~ 2.5 nm across, and in Class 4 there are 6 globular densities. No variation in ring thickness is present between the classes (Fig. 4A). Only the largest class (Class 4) shows significant occlusion of the central channel. This is more prominent on the face away from the grid. Pore size also does not appear to vary between the classes (measuring 2.5–3 nm across). The 3 smaller classes have a ring height of ~ 4 nm increasing to 5.5 nm for the larger class. The complexes are estimated to range from 70 kDa to 120 kDa based on the enclosed volume of the density maps at 3.8σ. In order to aid interpretation, we manually placed the published solution state NMR structure of TatAd monomers [37] into the density map of the largest (Class 4) TatAd complexes to give an indication of the relative sizes of the two structures (Fig. 4B). This comparison shows that the 3 nm long transmembrane helix (TMH) fits well into the ring density with the 4 nm long amphipathic helix (APH) forming a potential lid structure occluding the central cavity. Biochemical studies [38,39] have indicated that TatA homologues adopt a minimal tetrameric oligomeric state but placing 4 TatAd monomers into our density by no means fills the available density. We illustrate this point with our largest size class as insertion of the TatAd structure into the smaller classes would require some bending of the monomer to achieve a fit. Previous structural studies on TatAd using circular dichroism and solid-state NMR have shown that the APH is oriented parallel to the lipid bilayer [40–42], with the more recent solution state NMR study indicating a high level of flexibility in the APH C-terminal region [37]. This may explain the loss of lid structure density in the Class 2 and Class 3 density maps.

**Fig. 5:**
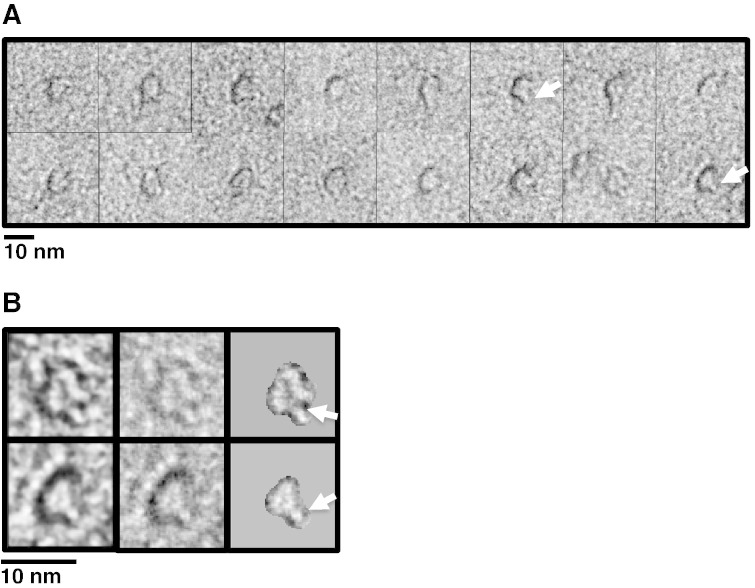
Small TatAd complexes show extensions from the ring. (A) TatAd particles were identified from the Class 1 tilted images showing one or two extensions (white arrows indicate examples) from the circumference of the ring measuring 1–2 nm in length. (B) Closer view of two such particles with and without a custom mask to highlight the extensions (white arrows).

The smallest class (Fig. 4A, Class 1) shows potential extensions arising from the globular ring densities. The clarity of the staining of these particles is somewhat affected in comparison with those in other classes, possibly due to the altered conformation. Alignment of these particles by projection matching showed that the ‘arm’ density was a significant feature of these particles and did not disappear during filtering. Manual analysis of the original tilted particles shows a range of small rings with these ‘arm’ extensions (Fig. 5A). One or two extensions measuring 1–2 nm can be seen to protrude from the circumference of the ring (Fig. 5A and B), and such extensions are not seen in the larger classes or in any of the untilted particles. The total length of these extended regions is ~ 4 nm including the ring density. A possible candidate for forming these extensions could be the APH of the TatAd subunit, which could form a structure compatible with an aqueous channel. Such a conformational change has been suggested previously for *E*. *coli* TatA [43] and could be due to the flexibility of the hinge region [37]. The flexibility of the hinge region may permit alternative arrangements of the APH, explaining why the ‘arm’ structure is seen in only a subset of TatAd complexes. This conformational change could explain the slight variation in ring diameter across the TatAd complexes observed, with the TMH shifting from a tilted orientation to a straighter alignment, parallel to the APH. However, it is also conceivable that the ‘arm’ density is due to other structural rearrangements within the Class1 TatAd complexes, for example as proposed in the recent ‘charge zipper mechanism’ for TatA function [44].

### 3.3 *B. subtilis* TatAc forms small, homogeneous complexes and can restore export of TorA in a ΔtatAE mutant

**Fig. 6:**
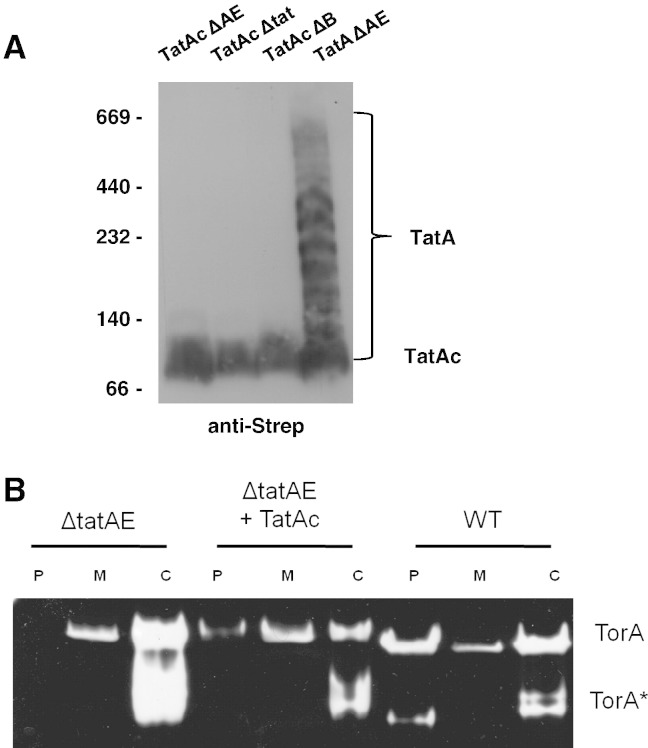
*B*. *subtilis* TatAc forms small homogeneous complexes, capable of complementing a *ΔtatAE E*. *coli* mutant. (A). Membranes were prepared from *E*. *coli ΔtatAE*, *Δtat*, and *ΔtatB* cells expressing *B*. *subtilis* TatAc, or from *E*. *coli ΔtatAE* cells expressing *E*. *coli* TatA. A *Strep*-II™ tag was present on the C-terminus of TatAc or TatA, respectively. The membranes were solubilised in digitonin and subjected to blue-native gel electrophoresis as described in the Experimental procedures section. The gel was immunoblotted with antibodies to the *Strep*-II™ tag. Mobilities of molecular mass markers (in kDa) are indicated on the left. Mobilities of TatAc and TatA complexes are also indicated. (B). The figure shows a native polyacrylamide gel stained for TMAO reductase (TorA) activity. Periplasm, membrane and cytoplasm samples (*P*, *M*, and *C* respectively) were prepared and analysed from *E*. *coli ΔtatAE* cells, from the same cells expressing *B*. *subtilis* TatAc from plasmid pBAD-Ac-Strep (ΔtatAE + TatAc), and from wild-type (WT) *E*. *coli* MC4100 cells. The mobility of active TorA is indicated. TorA* indicates a faster migrating form of TorA.

Our data on TatAd complexes represent the first structural information on the Tat complexes from a Gram-positive organism. The data show that the TatAd complexes are relatively small and structurally homogeneous, and this raises the question as to whether this is a general feature of such complexes. We have made a first step towards addressing this question by analysing another TatA-type complex from *B*. *subtilis*: the TatAc complex. We first expressed TatAc with a C-terminal Strep-II™ tag in *E*. *coli* ∆*tatAE and* ∆*tatB* cells. These were fractionated and the whole membranes were solubilised in 2% digitonin. The solubilised proteins were then analysed by blue native (BN) polyacrylamide gel electrophoresis to identify membrane protein complexes. The native gels were then subjected to immunoblotting with antibodies against the Strep-II™ tag. As shown in Fig. 6A, TatAc forms a small and homogeneous complex of ~ 100 kDa, similar to the complex observed for *E*. *coli* TatE [12], in the absence of other TatA-type complexes or TatB components. We also expressed TatAc in *E*. *coli* ∆*tat* cells and observed the same TatAc complex. This shows that the stability of the TatAc complex is neither affected by native expression levels of other *E*. *coli* Tat components, nor their absence. As with TatAd, the properties of this homogeneous TatAc complex contrast with the widely varying sizes of *E*. *coli* TatA bands observed with BN gels. Further to this, we tested whether TatAc can functionally replace TatA and TatE and restore translocation activity in *E*. *coli ΔtatAE* cells. The TMAO reductase activity assay was performed as described previously [12]. As shown in Fig. 6B, the expression of TatAc is sufficient to restore translocation activity in the presence of native levels of *E*. *coli* TatB and TatC, with no activity detectable in the periplasmic fraction in the absence of a TatA-like component (*ΔtatAE* control). The periplasmic signal with TatAc complementation (*ΔtatAE* + TatAc cells) is lower than in wild type cells, raising the possibility that translocation is less efficient. Nonetheless, the ability of TatAc to substitute for TatA/E provides strong evidence that the homogeneous TatAc complexes are physiologically relevant (Table 1).

## 4 Discussion

Previous studies have focussed on the structures of Tat complexes from the Gram-negative bacterium *E*. *coli* [12,18]. Very recently, the crystal structure of TatC from the hyperthermophilic bacterium *Aquifex aeolicus* was elucidated at 3.5 Å resolution [17]. These studies have prompted discussion concerning the possible mechanism of the Tat system with a particular emphasis on the roles of separate TatA and TatBC complexes during the actual translocation event. In contrast, Tat complexes from Gram-positive bacteria have not been subjected to structural analysis and, to date, only the structure of the *B*. *subtilis* TatAd monomer has been solved by solution NMR [37]. Furthermore, bioinformatic structural predictions of the TatCd monomer have been confirmed by circular dichroism [45]. Important biochemical differences have been noted between Tat complexes from Gram-positive and Gram-negative bacteria. Therefore, we have set out to investigate the structure of *B*. *subtilis* TatAd complexes through the generation of 2D and 3D structure models using EM techniques.

We show that TatAd forms ring-shaped structures of approximately 7.5–9 nm diameter, containing a potential pore of 2.5–3 nm, which is occluded at one end. Using electron microscopy and single particle analysis, we have obtained a 3D structure for the TatAd complex. This map reveals a pore, or well, which is occluded on one side of the membrane by a lid-like structure. We speculate that this lid comprises the amphipathic helices and C-terminal domains of the TatAd molecules, because these regions represent their most flexible parts [37]. The functional significance of such a ‘lid’ structure remains to be elucidated. A subset of the identified TatA complexes contain potential pores of diameters that could allow translocation of small Tat substrates and in this scenario the lid could seal a defined translocation channel at one end. On the other hand, our TatAd data argue against a ‘simple’ channel role for TatA-type complexes, because the diameters are too small to allow the passage of larger substrates, such as *E*. *coli* TorA which is 90 kDa in size. Notably, in the absence of clear data on how the small TatAd complexes could contribute to translocation, the role of the potential lid is unclear. Further experimentation is also required to accurately assess the protein contribution to the TatAd 3D density maps. The correlation of the presence of central density with a complex size suggests a mixed population of conformational states. In such a case the arm-like extensions seen extruding from the smallest class could likely include the C-terminus of the amphipathic helix of the TatAd subunit.

Previous analysis of *E*. *coli* TatA and TatE complexes revealed a similar structure with a prominent potential pore in the centre of the complexes [12,18]. Nevertheless, the two types of complexes exhibit major differences in other respects; TatA complexes are found in a large size range (9–13 nm diameter), whereas TatE complexes are much smaller and more homogeneous (6–8 nm diameter). The diameters of the potential pores are correspondingly different, and this has major implications for models of the translocation mechanism. The diameters of the potential pores in TatA complexes vary from 3 to 7 nm [12] and this range of pores would be capable of translocating the wide range of known Tat substrates in a folded state. Intriguingly, the largest pores would be just capable of accommodating larger Tat substrates, such as TorA. However, TatE is capable of substituting for TatA [46], although the structures of TatE complexes are very different to those of TatA complexes [12]. Importantly, the much smaller potential pores in TatE complexes are too small to accommodate many of the known Tat substrates. It was therefore suggested that the Tat mechanism may instead involve the coalescence of relatively homogeneous TatBC and TatA/E complexes, with the formation of a more flexible pore whose diameter adjusts according to the substrate being transported. In this context, our present TatAd complex data are of interest because, like *E*. *coli* TatE, TatAd is fully capable of substituting for *E*. *coli* TatA [24]. This implies that the TatAd complexes can interact with the native *E*. *coli* TatBC complex. In fact, the TatAd complexes resemble TatE complexes in that TatAd complexes do not present a large size range, and the average TatAd particle diameter (8 nm) is smaller than that of the smallest observed class of TatA complexes. Importantly, we do not observe TatAd complexes with an internal channel large enough for the translocation of fully folded *E*. *coli* Tat substrates.

Finally, the small and uniform size of the TatAd complex appears to be a strictly conserved feature of TatA complexes from *B*. *subtilis* as is underscored by our present investigation of the TatAc complex and our previous investigations of the TatAy complex [25,26]. We have therefore shown that all three TatA-type complexes of this Gram-positive bacterium form small homogenous complexes after purification. This challenges previous models on the role of TatA in protein translocation, which are based, in the main, on the biochemical and structural properties of *E*. *coli* TatA. Altogether, our observations emphasise the need for further functional studies to understand the nature and role of TatA-type complexes in the overall Tat mechanism for protein traffic.

## Figures and Tables

**Table 1:** Plasmids and strains used in this work.

Plasmids/cells	Relevant properties	Reference/source
pBAd-his	pBAD24 derivative containing the *B*. *subtilis tatAd*-*his* gene; Amp^r^	[21]
MC4100	F^−^*ΔlacU169 araD139 rpsL150 relA ptsF rbs flbB5301*	[36]
MC4100 *ΔtatABCDE*	*tat* deletion strain	[8]
MC4100 *ΔtatAE*	*tatA* and *tatE* deletion strain	[8]
MC4100 *ΔtatB*	*tatB* deletion strain	[8]
MC4100 *ΔtatC*	*tatC* deletion strain	[8]
pBAD-A-Strep	pBAD24 derivative containing the *E*. *coli tatA* gene with a C-terminal *Stre*p-II™ tag; Amp^r^	[37]
pBAD-Ac-Strep	pBAD24 derivative containing the *B*. *subtilis tatAc*-strepII gene; Amp^r^	This study

Amp^r^ = ampicillin resistant.
